# Long-term effects of an in-hospital program on sepsis management in the ICU

**DOI:** 10.1186/cc9633

**Published:** 2011-03-11

**Authors:** E Ferrari, G Serafini, D Trevisan, L Donno, L Rinaldi, M Girardis

**Affiliations:** 1 Università degli Studi di Modena e Reggio Emilia, Modena, Italy; 2 Policlinico di Modena, Italy

## Introduction

A hospital program named Sopravvivere alla Sepsi nel Policlinico di Modena http://www.policlinico.mo.it started in 2005 with the main objective to improve the survival rate of septic patients by means of continuous education and implementation of a sepsis operative protocol including the activation of a specific consultation by an intensivist and an infectious disease specialist. The aim of this study was to evaluate the long-term effects of this in-hospital program on compliance to treatments indicated by the evidence-based guidelines and on outcome in patients admitted to the ICU with septic shock (SS).

## Methods

In patients admitted with SS to a 10-bed ICU from January 2005 to December 2009 we collected: age, type of admission (medical or surgical), site of infection, SAPS II, 30-day mortality and the application of five resuscitative (blood cultures before antibiotics, antibiotics within 3 hours, source control, adequate fluid resuscitation, SvO_2 _optimization within 6 hours) and four management interventions (glycemia control, steroid use, rhAPC administration and plateau inspiratory pressure <30 cmH_2_O) as suggested by the surviving sepsis guidelines. Patients with end-stage liver disease, age <18 years and indications for end-of-life treatment were excluded.

## Results

A total of 129 patients have been evaluated and the number of SS admissions increased from a mean value of 19 patients/year in the period 2005 to 2007 to a value of 36 patients/year in the past 2 years. Age, SAPS II and site of infection were similar throughout the analyzed period whereas the percentage of medical admission increased from 33% to 42% in the past 2 years. Compliance to the five resuscitative interventions improved progressively from 24% in 2005 to 63% in 2007. Subsequently, they came back to values observed at the starting of the project (21% in 2008 and 25% in 2009). Similarly, the adherence to management interventions increased quickly after 2005 (from 14% to 50% in 2006) but decreased to a mean value of 35% in the past 3 years. Immediately after 2005, the observed 30-day mortality rate became lower than that predicted by the SAPS II, but it slightly increased from 31% in 2006 to 48% in 2009.

## Conclusions

The effects of an in-hospital program devoted to severe sepsis and SS management allowed an increase of ICU admissions for sepsis, a better management and an improvement of patients' survival rate. However, as expected, the adherence to guidelines gradually worsened with a slight increased in mortality rate in the past 2 years.

